# Sustainable Protein Sources: Functional Analysis of *Tenebrio molitor* Hydrolysates and Attitudes of Consumers in Poland and Spain Toward Insect-Based Foods

**DOI:** 10.3390/foods14020333

**Published:** 2025-01-20

**Authors:** Marcelina Maciejewska, Anna Dąbrowska, Marina Cano-Lamadrid

**Affiliations:** 1 Department of Development Functional Food Products, Wrocław University of Environmental and Life Sciences, 50-366 Wrocław, Poland; anna.dabrowska@upwr.edu.pl; 2 Department of Agri-Food Technology, Miguel Hernández University of Elche, Crta. de Beniel, Km. 3,2, 03312 Orihuela, Spain; marina.canol@umh.es

**Keywords:** edible insects, *Tenebrio molitor*, *Cucurbita ficifolia*, protein hydrolysis, consumer acceptance, functional foods

## Abstract

This study explores the potential of *Tenebrio molitor* protein hydrolysates as functional food ingredients, evaluating their bioactivity and consumer acceptance of the incorporation of edible insects into food across Poland and Spain. By aligning technical advancements with consumer preferences, this research bridges the gap between laboratory innovation and market feasibility, contributing to the development of sustainable functional foods. The study optimized the process of enzyme hydrolysis using serine protease from *Cucurbita ficifolia*, thereby enhancing DPPH scavenging capacity increased from 3.15 ± 0.53 to 8.17 ± 0.62 µM Trolox/mL and ABTS decolorization capacity increased from 4.29 ± 0.01 to 10.29 ± 0.01 µM Trolox/mL after 5 h of hydrolysis. Consumer surveys incorporating the Food Neophobia, Insect Phobia, and Entomophagy Scales revealed demographic and cultural influences on entomophagy acceptance. Among respondents, 27.1% in Poland and 25.7% in Spain had previously consumed insect-based products, while Polish participants showed a higher willingness to adopt insect-enriched foods. The study confirmed that hydrolysis enhances the antioxidant activity of *T. molitor* protein hydrolysates and that demographic and cultural factors significantly influence consumer acceptance of insect-based foods.

## 1. Introduction

The expanding global population causes a constantly growing demand for protein supply, the production of which is likely affecting the environment in severely negative ways. For example, CO_2_ emission based on cattle alone can lead to the release of 7.1 gigatons annually. By the year 2050, an increase of 75% is projected in meat consumption, which is a major contributor to environmental degradation. Therefore, it is crucial to reconsider the current food practices to ensure a more sustainable future. Recommendations to address climate change and environmental issues include reducing meat consumption, improving agricultural efficiency, and exploring sustainable protein sources. Environmentally friendly sources of protein, such as soybeans, legumes, nuts, seeds, whole grains, algae biomass, and insects are gaining prominence. Insects, as an alternative protein source, are promising in addressing the environmental issues associated with meat production as they have the potential to reduce greenhouse gas emissions [1,2,3].

Insects have long been a food source across various cultures, particularly in Africa Asia, Latin America, and Australia, although Western societies view insect consumption with skepticism. Cultural perceptions of disgust are a barrier, requiring strategies to promote awareness of the nutritional and environmental benefits insects offer [4,5]. Studies show varying levels of food neophobia and neophilia across Europe. While food neophobia may act as a barrier to the acceptance of insect-based foods, food neophilia can equally drive curiosity and openness to try novel and unfamiliar products, creating the potential for the gradual adoption of such foods [6]. As Ghosh et al. point out, the acceptance or rejection of edible insects is influenced by multiple factors, including cultural traditions, sensory attributes (such as taste and texture), and the availability of insects as a food source. They emphasize that strategies to promote the acceptance of insect-based foods must address both cultural perceptions and sensory experiences, highlighting the importance of positive exposure to such foods to overcome barriers of disgust and unfamiliarity [7].

The European Food Safety Authority (EFSA) has approved several insect species, including *Tenebrio molitor* (yellow mealworm), as novel foods. However, protein hydrolysates derived from mealworms have not yet been authorized for human consumption. Known for its rich protein profile and essential amino acids like leucine and lysine, *T. molitor* is considered one of the most promising insect-based foods [8,9]. Research into food neophobia underscores the importance of understanding cultural differences when introducing insects as a novel food source [10,11].

*Tenebrio molitor*, or yellow mealworm, offers a high protein content (41–66%) and a mild flavor profile, including nutty and umami notes. It has a well-established farming system, primarily for fish and bird feed, which could support its expanded use in human diets [12,13]. As a source of bioactive peptides, mealworms also show health benefits such as antioxidant and antimicrobial effects, making them suitable for sustainable, nutritious foods [14,15]. Additionally, Ghosh et al. highlighted that *Tenebrio molitor* has a high-fat content (34.5%) and a protein profile that meets all essential amino acid requirements recommended by FAO/WHO/UNU [16]. This combination of high protein quality and lipid content positions mealworms as a nutritionally dense and versatile ingredient for future food systems. In order to fully benefit from the nutritional potential of *Tenebrio molitor*, in particular, its high protein and bioactive peptide content, it is essential to use methods that optimize its functional properties.

Enzymatic hydrolysis is an efficient and gentle process that extracts bioactive peptides from protein sources, retaining nutritional value and enhancing functional properties. This method allows for control over product composition, including amino acid profiles and molecular weights, making it ideal for developing protein-rich diets [17]. The demand for new, cost-effective enzymes has led to the exploration of unconventional enzymes, including those from *Cucurbita ficifolia.*

Serine protease from *C. ficifolia* is a promising unconventional enzyme due to its specific proteolytic properties, which target proteins like casein, gluten, and ovalbumin. When applied to whey proteins and αs-casein in cow’s milk, this protease has been shown to significantly reduce immunoreactivity to IgE and IgG, suggesting potential applications in allergy prevention, especially for infants at high allergy risk [17,18,19,20,21].

The unique properties of serine protease from *C. ficifolia* support its application in developing hydrolysates and enhancing the nutritional profiles of various protein sources. This enzyme, with its effective and affordable production, also contributes to expanding enzyme options in food production and diversifying protein hydrolysates, which include free amino acids, oligopeptides, and bioactive peptides with antioxidant activities.

This study evaluates the potential of edible insects as a sustainable alternative protein source. The bioactivity of *Tenebrio molitor* proteins is investigated following enzymatic hydrolysis using non-commercial serine proteases derived from *Cucurbita ficifolia*. In this study, bioactivity refers to the biological properties of *Tenebrio molitor* protein hydrolysates, particularly their antioxidant potential, which was evaluated using DPPH-free radical scavenging and ABTS cation decolorization assays. These properties are indicative of the hydrolysates’ functional potential for use in health-promoting food products. Additionally, consumer acceptance of new food products, particularly edible insects, is assessed through a survey of Polish and Spanish consumers. The survey employed the Food Neophobia Scale (FNS), the Insect Phobia Scale (INS), and the Entomophagy Scale (ENS). Moreover, the survey evaluated consumers’ willingness to incorporate insects into other food matrices and their preferences based on the types and sensory properties of insect-enriched products.

## 2. Materials and Methods

### 2.1. Materials

The larvae of yellow mealworm (*Tenebrio molitor*) were sourced from TENEBRIA Ltd. (Lubawa, Poland). The larvae were frozen at −80 °C for 24 h, followed by sublimation drying for an additional 24 h at 40 °C under 1.650 mbar pressure. After drying, lipid extraction was performed using supercritical fluid extraction (SFE) with carbon dioxide at 30 MPa pressure and 60 °C temperature. This process resulted in defatted yellow mealworm flour, which was stored at −80 °C until further use. The serine protease enzyme was extracted from Asian pumpkin (*Cucurbita ficifolia*) following the methodology described by Dryjański et al. [22]. All reagents and chemicals used in this study were of analytical grade and were procured from standard suppliers.

### 2.2. Experimental Design

The study comprised two interconnected phases. In the initial phase, the focus was on optimizing the enzymatic hydrolysis of proteins from defatted yellow mealworm flour to enhance their functional properties, particularly their antioxidant activity. In the subsequent phase, consumer attitudes and preferences toward insect-based foods were evaluated through a comprehensive survey among Polish and Spanish participants.

### 2.3. Hydrolysis of Proteins

The proteolytic activity against casein was quantified in accordance with the methodologies delineated by Chrzanowska and Koaczkowska [23]. A 0.5% (*w*/*v*) protein solution was prepared by suspending defatted yellow mealworm flour in distilled water, with the pH adjusted to 4.5. The suspension was then homogenized using a blender and subjected to centrifugation at 8000 rpm to separate the plasma. This process was repeated after subjecting the suspension to a freezing and drying cycle to ensure consistent protein extraction. Enzymatic hydrolysis was then carried out using serine protease at a dose of 300 U/mg of hydrolyzed protein in a 0.1 M Tris-HCl buffer (pH 8.0). The hydrolysis reactions were carried out at temperatures of 37 °C for durations of 1, 3, and 5 h, with the reaction being terminated by thermal inactivation at 90 °C. The protein content was determined using the Lowry method [24]. The degree of hydrolysis (DH) was then quantified based on the content of free amino groups (FAG), with trinitrobenzene sulfonic acid (TNBS) being used as described by Snyder and Sobocinski and Kuchroo and Ramilly [25,26]. Subsequently, the hydrolyzed protein fractions were subjected to SDS-PAGE under denaturing conditions [27].

### 2.4. Determination of Antioxidant Activity

Antioxidant activity was assessed using two standard methods: DPPH radical scavenging activity and ABTS cation decolorization assay. The DPPH assay was conducted in accordance with the methodology described by Kedare and Singh [28], while the ABTS assay adhered to the procedure outlined by Re et al. [29]. Both methods were applied to hydrolysates obtained after 1, 3, and 5 h of hydrolysis, as well as to fully hydrolyzed samples, to determine their antioxidant potential at different stages of hydrolysis.

### 2.5. Consumer Survey

An English questionnaire was designed by the authors to address the objectives of this study. It was then translated into Polish and Spanish to ensure linguistic consistency and cultural relevance for the respondents in both countries. Google Forms was used as the platform for data collection. Ethical approval was obtained from the UMH Institutional Review Board prior to the distribution of the questionnaire (ref: DTA.MCL.2240123). The questionnaire received a total of 140 responses (n = 140), with 70 respondents from each of the targeted countries (Poland and Spain). It was structured into sections, covering demographics, consumer behavior, and consumer preferences. Participants’ responses were evaluated on a hedonic scale from 1 to 7, with 1 indicating complete disagreement and 7 indicating complete agreement with the statements [30,31,32,33,34]. The consumer survey was conducted to understand consumer attitudes and preferences, ensuring that edible insect-based products align with market needs. Psychometric tools provided insights into factors influencing acceptance, bridging the gap between innovation and consumer demand. The consumer survey focused on the general acceptance of insect-based foods to explore cultural and demographic influences. Specific questions about *T. molitor* protein hydrolysates were avoided to ensure reliability, as respondents were unlikely to have prior familiarity. This foundational approach informs future studies on hydrolysate applications.

### 2.6. Statistical Analyses

The statistical analysis of the study was conducted using Excel and RStudio R 4.4.2. The analysis began with a one-way analysis of variance (ANOVA) to identify statistically significant differences in the means of the data. This was followed by a Tukey’s post hoc test for multiple comparisons. All laboratory experiments were carried out in triplicate (n = 3), and a *p*-value threshold was used for significance (*p* < 0.05). The resulting data are expressed as the mean ± standard deviation (SD). The consumer survey data analysis followed the methodological framework described by Fernández-Ruiz et al., which included reliability assessments and mixed model ANOVA to analyze relationships between food neophobia and consumer preferences. Laboratory analyses, including protein hydrolysis and antioxidant activity determinations, were conducted in accordance with the methodological framework established by Szołtysik et al., thereby ensuring the reliability and replicability of the results [35,36].

## 3. Results and Discussion

### 3.1. Determination of Antioxidant Activity of Hydrolysates

**Figure 1:**
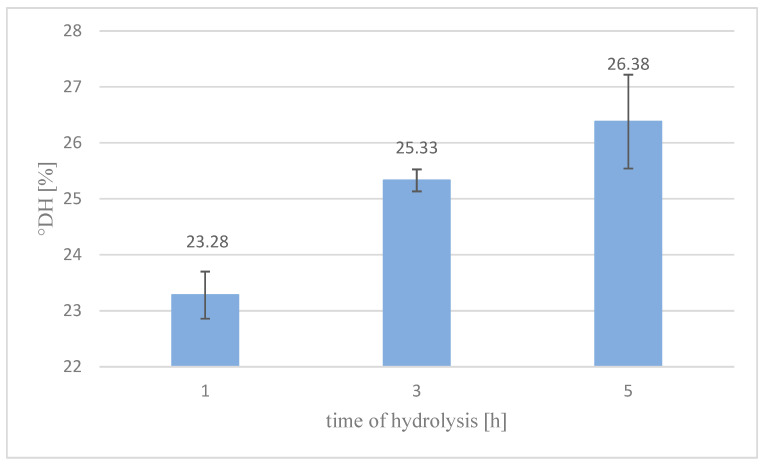
Degree of hydrolysis [%] obtained with the use of serine protease from Asian pumpkin at a dose of 300 U/mL protein.

**Figure 2:**
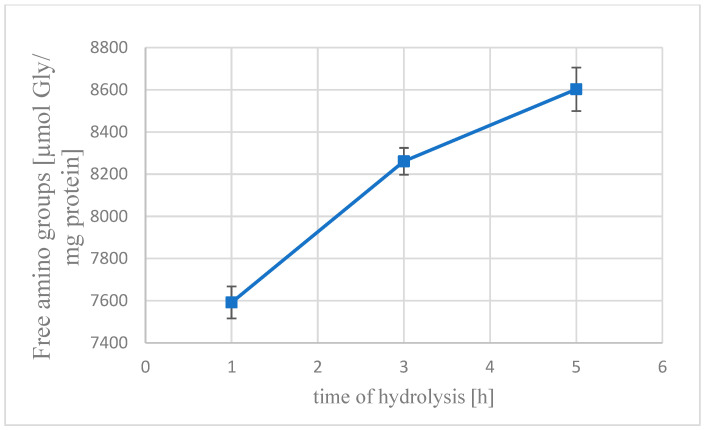
Increase in free amino groups [µmol Gly/mg protein] in hydrolysate.

The results presented in Figure 1 were obtained from both methods and indicate that an increase in the degree of hydrolysis occurs with an increase in hydrolysis time. The highest values (26.38%) were obtained after 5 h of degradation. As expected, the degree of hydrolysis increased proportionally with hydrolysis time, consistent with well-established biochemical principles. Consequently, this finding substantiates the efficacy of *Cucurbita ficifolia* protease in hydrolyzing defatted yellow mealworm protein within the experimental parameters. The enzymatic hydrolysis process led to a progressive increase in the degree of hydrolysis [%] as shown in Figure 2. Notably, the highest free amino group (FAG) concentration was observed after a five-hour reaction, reaching 8602.84 [µmol Gly/g protein], with the total hydrolysis score culminating at 32,608.1 [µmol Gly/g protein]. This result highlights the efficiency of the hydrolysis process over time, demonstrating a direct correlation between the reaction duration and protein degradation, as evidenced by the measurable increase in FAG levels.

SDS-PAGE analysis under denaturing conditions confirmed protein degradation over time. The control sample showed intact high molecular weight insect proteins. After 1 h of hydrolysis, the protein fraction partially degraded, forming peptides of 25 to 35–40 kDa, indicated by additional lower molecular weight bands and reduced intensity of original bands. Further hydrolysis (3 and 5 h) showed similar degradation. The deepest degradation occurred within the first hour, aligning with the increase in the degree of hydrolysis and FAG levels, demonstrating the efficiency of the hydrolysis process.

Following enzymatic hydrolysis with serine protease, the antioxidant activity of the hydrolysates was analyzed to evaluate the functional impact of the process. The results, presented in Table 1, highlight the effectiveness of the hydrolysis process in enhancing antioxidant activity, measured through DPPH scavenging and ABTS decolorization, with extended hydrolysis times. The 1 h hydrolysate shows moderate antioxidant activity, which further improves in the 3 h and 5 h samples. The highest antioxidant activity is observed in the fully hydrolyzed sample, suggesting that prolonged hydrolysis enhances the release of antioxidant peptides. This pattern aligns with previous studies showing that insect protein hydrolysates exhibit significant antioxidant potential. Studies highlight that hydrolysates, particularly those produced with alcalase enzymes, show enhanced antioxidant and hepatoprotective effects compared to raw powder. These enhancements render hydrolysates highly suitable for functional food applications, where superior solubility, digestibility, and bioactivity are paramount. Previous studies have demonstrated the antioxidant potential of insect protein hydrolysates, which is attributed to the release of bioactive peptides during enzymatic hydrolysis. These peptides have shown significant antioxidant and hepatoprotective effects, highlighting their suitability for functional food applications [37,38].

### 3.2. Consumer Survey

Table 2 provides the demographic breakdown of the consumer survey among Spanish and Polish respondents. The sample consists of more females in both groups. Most Polish respondents fall within the 18–25 age range, while the Spanish cohort is more evenly distributed across age groups, particularly in the 26–35 and 46–55 brackets. These demographic differences influence perceptions of entomophagy. Men are generally more inclined to try unprocessed insects, whereas processed insect products show a more balanced acceptance across genders [5]. The younger Polish demographic, with a higher percentage of females, suggests that targeting younger female consumers might be an effective approach. In contrast, the broader age range in Spain indicates a wider target demographic.

Studies by Mustapa and Kallas and Ordoñez López et al. indicate that food preferences tend to solidify with age, leading to higher levels of food neophobia among middle-aged respondents due to established eating habits [39,40]. Education levels also differ significantly, with more Polish respondents having only a high school education and an equal number holding bachelor’s degrees, while Spanish respondents show higher levels of postgraduate education (32.9% Ph.D. and 30% master’s degrees). Most Polish consumers live in large cities, whereas Spanish consumers are more dispersed, with nearly 25% residing in villages and over 20% in small cities. The higher educational levels among Spanish participants may increase their openness to novel foods, such as insect-based products, due to greater awareness of their benefits [5].

Insect-based foods show generally low engagement among Polish and Spanish consumers, as shown in Table 3, with only a small portion having consciously tried insects or insect-enriched products. This limited exposure reflects a broader trend of food neophobia and cultural hesitance toward novel foods, particularly insect-based products. Such barriers are well-documented in research, which emphasizes that unfamiliarity and cultural perceptions strongly influence consumer acceptance. Addressing these factors through awareness campaigns focused on the nutritional and environmental benefits of insect-based foods could help mitigate neophobic attitudes and increase willingness to try these products [41].

Consumer willingness to try insect-based products, influenced by various factors, is illustrated in Table 4, with Spanish respondents generally showing a higher baseline acceptance than Polish respondents. Both groups show increased willingness when informed about the nutritional benefits and assured of the safety of insect-based products, highlighting the importance of educational efforts. Familiarity and knowledge about edible insects, as indicated by prior studies, play a critical role in consumer openness. Emphasizing nutritional and safety aspects in targeted marketing could therefore boost acceptance, particularly in Poland, where these assurances seem to significantly impact consumer willingness [32,42].

Table 5 illustrates the distribution of attitudes toward entomophagy, with Spanish respondents showing a higher prevalence of neophilic attitudes compared to Polish respondents, while most individuals in both groups display neutral attitudes. This pattern aligns with existing research, which identifies food neophobia as a barrier to the acceptance of insect-based foods. Studies indicate that neophobic tendencies, often linked to factors such as age and education, reduce the willingness to try novel foods, including insect-based options, especially among older and less-educated individuals. The high internal consistency of the FNS, INS, and ENS scales confirms their reliability in assessing these attitudes. Targeted educational and marketing strategies that emphasize the safety, nutritional benefits, and environmental advantages of insect-based products could effectively reduce food and insect phobia, thereby enhancing acceptance [40].

Differences in attitudes between Polish and Spanish consumers regarding the incorporation of insects into other food matrices, especially in animal products, are presented in Table 6. Polish respondents generally show a stronger acceptance, finding the idea of consuming insect-fed animal products more natural and beneficial for environmental sustainability. In contrast, Spanish respondents exhibit lower acceptance, with some viewing insect-fed animal products as less palatable or even unappealing. Both groups, however, recognize the potential environmental benefits of insect incorporation in the food chain. These findings suggest that framing insect-fed products within an environmental and sustainability context could help increase consumer openness, especially in populations that currently display more reservations.

**Figure 3:**
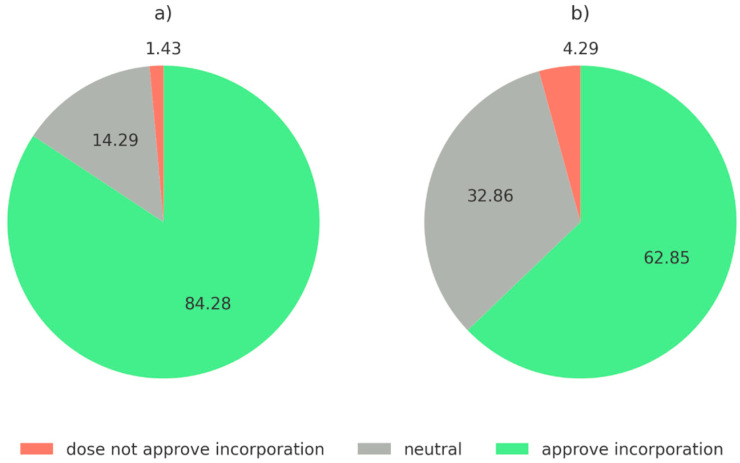
Spanish and Polish consumer acceptance of insect’s incorporation in food matrix [%]: (**a**) Polish consumer responses. (**b**) Spanish consumer responses.

Figure 3 illustrates a higher overall acceptance among Polish consumers for incorporating insects into food matrices compared to Spanish consumers. The stronger acceptance in Poland suggests a greater openness to insect-based innovations, likely influenced by younger demographics and a more urbanized population. Spanish consumers’ reluctance may stem from cultural factors such as unfamiliarity with insect-based products and potential disgust associated with insects as food sources. Addressing these cultural and psychological barriers through targeted marketing and educational campaigns could foster greater acceptance by highlighting the benefits of insect-based food production, such as sustainability and reduced environmental impact.

**Figure 4:**
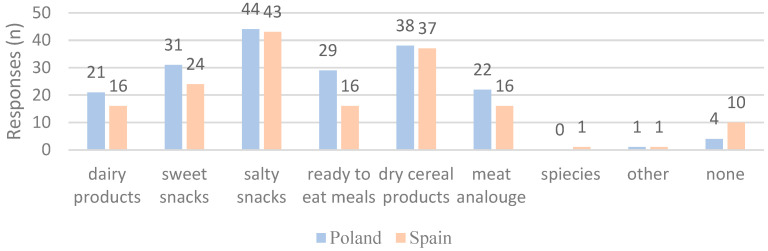
Consumer preferences about products enhanced with insect powder.

A comparative analysis of consumer preferences for products enhanced with insect powder among Polish and Spanish respondents (Figure 4) reveals significant differences and shared trends. Both groups show a clear preference for salty snacks and dry cereal products, with Polish consumers exhibiting a more pronounced inclination toward these categories. Additionally, Polish respondents demonstrate greater interest in sweet snacks and ready-to-eat meals, which may reflect differences in taste, food traditions, and food availability. In contrast, Spanish respondents indicate greater reluctance toward insect-enhanced products, highlighting cultural and demographic influences on acceptance.

**Figure 5:**
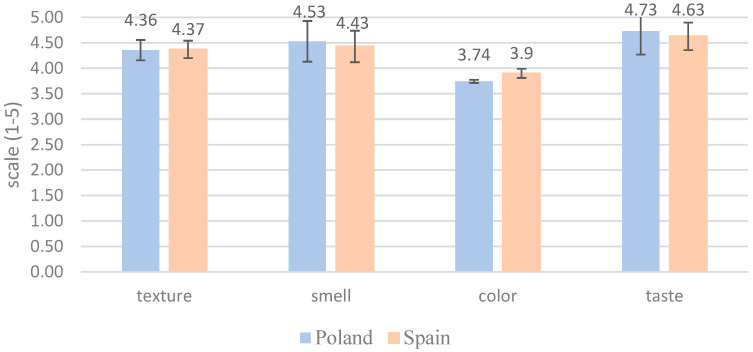
Consumer Perceptions of Sensory Aspects in Insect-Enhanced Foods (0—Completely Irrelevant, 5—Highly Relevant).

The relevance of different sensory aspects for consumers regarding insect-enhanced food products is considered. It is found that texture, smell, and taste are of greater importance than color (Figure 5).

When evaluating the relevance of sensory aspects for insect-enhanced food products, both Polish and Spanish consumers prioritize texture, smell, and taste over color. This aligns with the findings of the study by Martins et al., which emphasize the importance of sensory experiences in consumer acceptance of novel foods, particularly insect-based products [42].

## 4. Conclusions

This study investigated the functional properties of *Tenebrio molitor* protein hydrolysates, with a particular emphasis on their antioxidant activity and the consumer acceptance of insect-based foods. The null hypothesis—that enzymatic hydrolysis using Cucurbita ficifolia protease would enhance the bioactivity of yellow mealworm protein hydrolysates and that consumer acceptance would vary by demographic factors—was confirmed. The hydrolysis process was found to significantly enhance antioxidant activity, as evidenced by DPPH and ABTS assays. Additionally, the study revealed notable differences in consumer attitudes between Polish and Spanish populations.

From a scientific perspective, the findings demonstrate the effectiveness of *Cucurbita ficifolia* protease in producing bioactive peptides with enhanced antioxidant properties. This contributes to the growing body of research on non-commercial proteases and underscores the potential of yellow mealworm protein as a sustainable and functional ingredient for health-promoting foods. The observed correlation between hydrolysis efficiency and bioactivity supports the applicability of bioactive peptides in addressing oxidative stress, making them valuable for functional food formulations.

The consumer survey revealed that acceptance of insect-based foods is influenced by cultural and demographic factors. Younger and urbanized Polish consumers showed greater openness, while Spanish participants demonstrated cautious interest, likely shaped by established food habits and higher levels of education. These findings suggest that tailored marketing and educational strategies are essential to overcoming barriers to acceptance. For instance, emphasizing the sustainability and nutritional benefits of insect-based foods can appeal to environmentally conscious consumers, while addressing sensory concerns can mitigate reluctance.

In terms of practical applications, the study underscores the potential of mealworm hydrolysates as adaptable ingredients for incorporation into diverse food matrices. Their enhanced bioactivity, when combined with insights into consumer attitudes, provides a strong foundation for the development of innovative insect-based products that are aligned with health and sustainability trends. Future research should prioritize the establishment of links between consumer perceptions and specific product characteristics, such as hydrolysates, through targeted surveys and sensory evaluations. This integrative approach serves to fill the gap between technical advancements and market demands, providing a foundation for the successful adoption of insect-based functional foods.

## Figures and Tables

**Table 1:** Bioactivity values: antioxidant activity as the ability to scavenge DPPH free radicals and decolorize ABTS cations [µM Trolox/mL].

Sample	DPPH	ABTS
1H	3.15 ± 0.53	4.29 ± 0.01
3H	3.48 ± 0.07	4.99 ± 0.32
5H	3.65 ± 0.01	5.15 ± 0.02
total hydrolysis	8.17 ± 0.62	10.29 ± 0.01

**Table 2:** Demographic section results among Spanish and Polish consumers (*n* = 140).

Characteristic	Breakdown	Polish [%]	Spanish [%]
Gender	Female	70.0	65.7
	Male	30.0	34.3
Age	18–25	62.9	21.4
	26–35	22.9	28.6
	36–45	2.9	12.9
	46–55	7.1	15.7
	56–65	1.4	15.7
	>65	2.9	5.7
Education level	Less than High School	0.0	1.4
	High School	41.4	5.7
	Bachelor’s degree	41.4	28.6
	Master’s degree	14.3	30.0
	Ph.D.	1.4	32.9
	I prefer not to answer	1.4	1.4
City size	Village	21.4	24.3
	City up to 50 thousand residents	8.6	21.4
	City of 50–100 thousand residents	12.9	12.9
	City over 100 thousand residents	55.7	37.1
	I do not know	1.4	4.3

**Table 3:** Previous experience with the consumption of insects among Polish and Spanish consumers [n, %].

Questions		Poland		Spain	
		Yes	No	Yes	No
Have you ever conscientiously tasted insects as food?	[n]	19	51	18	52
	[%]	27.1	72.9	25.7	74.3
Have you ever conscientiously tasted food enriched with insects?	[n]	17	53	20	50
	[%]	24.3	75.7	28.6	71.4

**Table 4:** Willingness to consume insects among Polish and Spanish consumers [n, %].

Answers		Poland	Spain
Yes, of course	[n]	8	18
	[%]	11.4	25.7
Only if I were aware of beneficial nutritional value of those products	[n]	20	14
	[%]	28.6	20.0
Only if I would be sure about the safety of those products	[n]	20	15
	[%]	28.6	22.0
Only if there wouldn’t be noticeable change in taste of products	[n]	15	10
	[%]	21.4	14.0
No, never	[n]	7	13
	[%]	10	18

**Table 5:** Results of Food Neophobia, Insect Phobia, and Entomophagy Scales [n, %].

Food Neophobia Scale Results (FNS)			Insect Phobia Scale (INS)			Entomophagy Scale (ENS)		
	Poland	Spain		Poland	Spain		Poland	Spain
Neophilia	25.71	32.86	Insectophilia	12.86	24.29	Entomophilia	32.86	24.29
Neutral	71.43	61.43	Neutral	58.57	50.00	Neutral	33.00	51.43
Neophobia	2.86	5.71	Insectophobia	28.57	25.71	Entomophobia	20.00	24.29
Cronbach’s Alpha	0.78	0.79	Cronbach’s Alpha	0.89	0.81	Cronbach’s Alpha	0.91	0.90

**Table 6:** Mean scores and standard deviation [mean ± SD] of each question for incorporation of insects on a food matrix, where 1—completely disagree and 7—completely agree.

Questions	Poland	Spain
It seems natural to me that animals feed on insects	6.26 ± 1.08 ^a^	5.73 ± 1.53 ^b^
The idea of consuming meat/milk from animals fed on insects is not disgusting	5.54 ± 1.65 ^a^	4.66 ± 2.14 ^b^
I believe that consuming meat/milk from animals fed with insects-based feed is good for the environment	5.50 ± 1.39 ^a^	5.20 ± 1.64 ^a^
I believe there are no other ways to improve environmental sustainability in animal production than introducing insects	4.83 ± 1.59 ^a^	4.10 ± 1.83 ^b^
I would be willing to consume meat/milk from animals fed on insect meal as long as the food is not expensive	5.40 ± 1.54 ^a^	5.00 ± 2.01 ^a^

^a, b^: Diffrent letters indicate statistically significant differences between means at *p* < 0.05—Tukey’s post hoc test.

## Data Availability

The original contributions presented in the study are included in the article. Should further inquiries necessitate additional information, the corresponding author can be contacted directly.
